# Supplementary material for: Nationwide Screening for Bee Viruses in Apis mellifera Colonies in Egypt
Source: Insects. 2023 Feb 9;14(2):172. doi: 10.3390/insects14020172 (PMC9964814; doi:10.3390/insects14020172)
Supplement: Supplementary file 1 [file insects-14-00172-s001.zip › Supplementary materials_graphs_R1_RJP.pdf]

Supplementary materials

Figure S1.

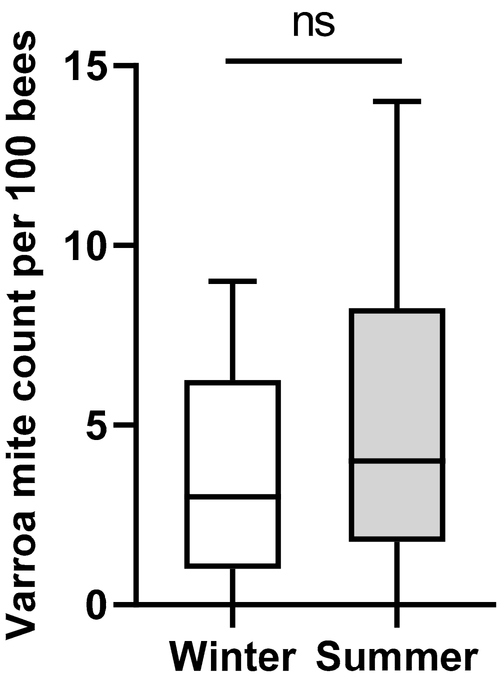

Figure S1. Varroa mite intensity of infestation in honey bee samples collected from 54 apiaries across 18 governorates in Egypt during winter and summer 2021. There was no significant difference in varroa mite count between seasons (Student's *t*-test, $P > 0.05$). Each boxplot shows the median and interquartiles while the whiskers show 95% confidence intervals.
